# Immune Responses in Acute and Convalescent Patients with Mild, Moderate and Severe Disease during the 2009 Influenza Pandemic in Norway

**DOI:** 10.1371/journal.pone.0143281

**Published:** 2015-11-25

**Authors:** Kristin G.-I. Mohn, Rebecca Jane Cox, Gro Tunheim, Jan Erik Berdal, Anna Germundsson Hauge, Åsne Jul-Larsen, Norwegian Pandemic Group, Bjoern Peters, Fredrik Oftung, Christine Monceyron Jonassen, Siri Mjaaland

**Affiliations:** 1 The Influenza Centre, Department of Clinical Science, University of Bergen, Bergen, Norway; 2 Infectious Diseases Unit, Department of Internal Medicine, Haukeland University Hospital, Bergen, Norway; 3 Department of Research & Development, Haukeland University Hospital, Bergen, Norway; 4 Division of Infectious Disease Control, Department of Bacteriology and Immunology, Norwegian Institute of Public Health, Oslo, Norway; 5 K.G. Jebsen Centre for Influenza Vaccine Research, Department of Clinical Science, University of Bergen, Bergen, and The Norwegian Institute of Public Health, Oslo, Norway; 6 Department of Infectious Diseases, Akershus University Hospital, Nordbyhagen, Norway; 7 Section for Virology, Department of Laboratory Services, Norwegian Veterinary Institute, Oslo, Norway; 8 Division of Infectious Disease Control, Norwegian Institute of Public Health, Oslo, Norway; 9 Division of Vaccine Discovery, La Jolla Institute for Allergy and Immunology, La Jolla, California, United States of America; 10 Genetic Unit, Department of Multidisciplinary Laboratory Medicine and Medical Biochemistry, Akershus University Hospital, Nordbyhagen, Norway; 11 Genetic Unit, Centre for Laboratory Medicine, Østfold Hospital Trust, Fredrikstad, Norway; 12 Department of Chemistry, Biotechnology and Food Science, Norwegian University of Life Sciences, Ås, Norway; Icahn School of Medicine at Mount Sinai, UNITED STATES

## Abstract

Increased understanding of immune responses influencing clinical severity during pandemic influenza infection is important for improved treatment and vaccine development. In this study we recruited 46 adult patients during the 2009 influenza pandemic and characterized humoral and cellular immune responses. Those included were either acute hospitalized or convalescent patients with different disease severities (mild, moderate or severe). In general, protective antibody responses increased with enhanced disease severity. In the acute patients, we found higher levels of TNF-α single-producing CD4^+^T-cells in the severely ill as compared to patients with moderate disease. Stimulation of peripheral blood mononuclear cells (PBMC) from a subset of acute patients with peptide T-cell epitopes showed significantly lower frequencies of influenza specific CD8^+^ compared with CD4^+^ IFN-γ T-cells in acute patients. Both T-cell subsets were predominantly directed against the envelope antigens (HA and NA). However, in the convalescent patients we found high levels of both CD4^+^ and CD8^+^ T-cells directed against conserved core antigens (NP, PA, PB, and M). The results indicate that the antigen targets recognized by the T-cell subsets may vary according to the phase of infection. The apparent low levels of cross-reactive CD8^+^ T-cells recognizing internal antigens in acute hospitalized patients suggest an important role for this T-cell subset in protective immunity against influenza.

## Introduction

During the 2009 influenza pandemic, young and otherwise healthy people experienced severe illness and mortality [1–4]. During the main wave of the pandemic in Norway, 1300 people were hospitalized, 200 patients received intensive care treatment, and 29 patients died [5]. Nevertheless, in hindsight, this pandemic was regarded as mild [6]. Post-pandemic studies have described the clinical picture, the risk factors associated with disease outcome, and effects of vaccines and antiviral medication [1,3,7–12]. Specific viral mutations and several host factors and underlying conditions, such as obesity and pregnancy, were identified and associated with increased disease severity [13–17]. People older than 65 years old experienced less severe infection, probably due to pre-existing cross-reactive immunity generated by previous H1N1 infections [18].

Seasonal vaccination or infection induces strain-specific neutralizing antibodies directed towards the viral surface glycoproteins, hemagglutinin (HA) and neuraminidase (NA). HA-specific antibodies measured by the hemagglutination inhibition assay (HI) are defined as the primary correlate of protection against influenza in man (HI titers ≥40) [19]. However, strain-specific antibodies do not provide cross-protection against new epidemic or pandemic viruses [20]. Hence, due to the lack of protective antibodies, the novel A(H1N1)pdm09 virus spread rapidly worldwide.

In contrast to antibodies, T-cells may mediate cross-protective immunity between strains due to recognition of epitopes from the conserved core antigens of the virus, which have a high degree of homology, e.g. (nucleoprotein (NP), the polymerases (PB1, PB2 and PA) and matrix (M) proteins. T-cells play important roles in coordinating and regulating the immune response against influenza [21]. CD4^+^ T-cells help B-cells in producing neutralizing antibodies and secrete cytokines, which direct the activity of CD8^+^ T-cells. CD8^+^ T-cells contribute to protection by killing virus-infected host cells, and are essential for viral clearance. Infection with seasonal influenza A H1N1 virus induces memory T-cells that cross-react with the pandemic strain [22–25]. In a recent study from the UK, the presence of NP-specific T-cells prior to exposure was associated with significantly less symptomatic, PCR-positive seasonal and pandemic influenza disease [25]. More specifically, pre-existence of CD8^+^ T-cells against conserved viral core epitopes correlated inversely with symptomatic illness in antibody naïve adults during the 2009 pandemic [26]. However in a human, high dose challenge model of seasonal influenza A virus, pre-existing influenza-specific CD4^+^ T-cells, rather than CD8^+^ T-cells, correlated with protection against mild disease [27]. In the early phase of A(H1N1)pdm09 virus infection, high levels of peripheral CD4^+^ T-cells may correlate with disease severity [28], and different immune memory profiles develop depending on the severity of pandemic infection [29].

In the absence of strain specific antibodies, cross-reactive T-cells are considered important, as cellular immune responses may limit disease severity and death when infection is already established [21]. Current knowledge of human T-cell responses after natural infection with influenza remains limited. Due to the sudden nature of pandemics, with a stretched healthcare system primarily focused on treatment, there is limited immunological data from hospitalized patients with different disease severities [30]. Here, we describe and compare the immune responses in acute and convalescent patients with different pandemic disease severities, with the hypothesis that the severely ill patients would have less cross-protective T-cell immunity. Although our study has limitations in sample sets and study design imposed by the pandemic, our results suggest that both the antigen targets and the T-cell subsets involved in recognition vary according to the phase of infection. This study increases our understanding of the immune responses associated with severe disease and hospitalization and may guide future treatment and development of improved influenza vaccines.

## Material and Methods

### Study design

**Fig 1:**
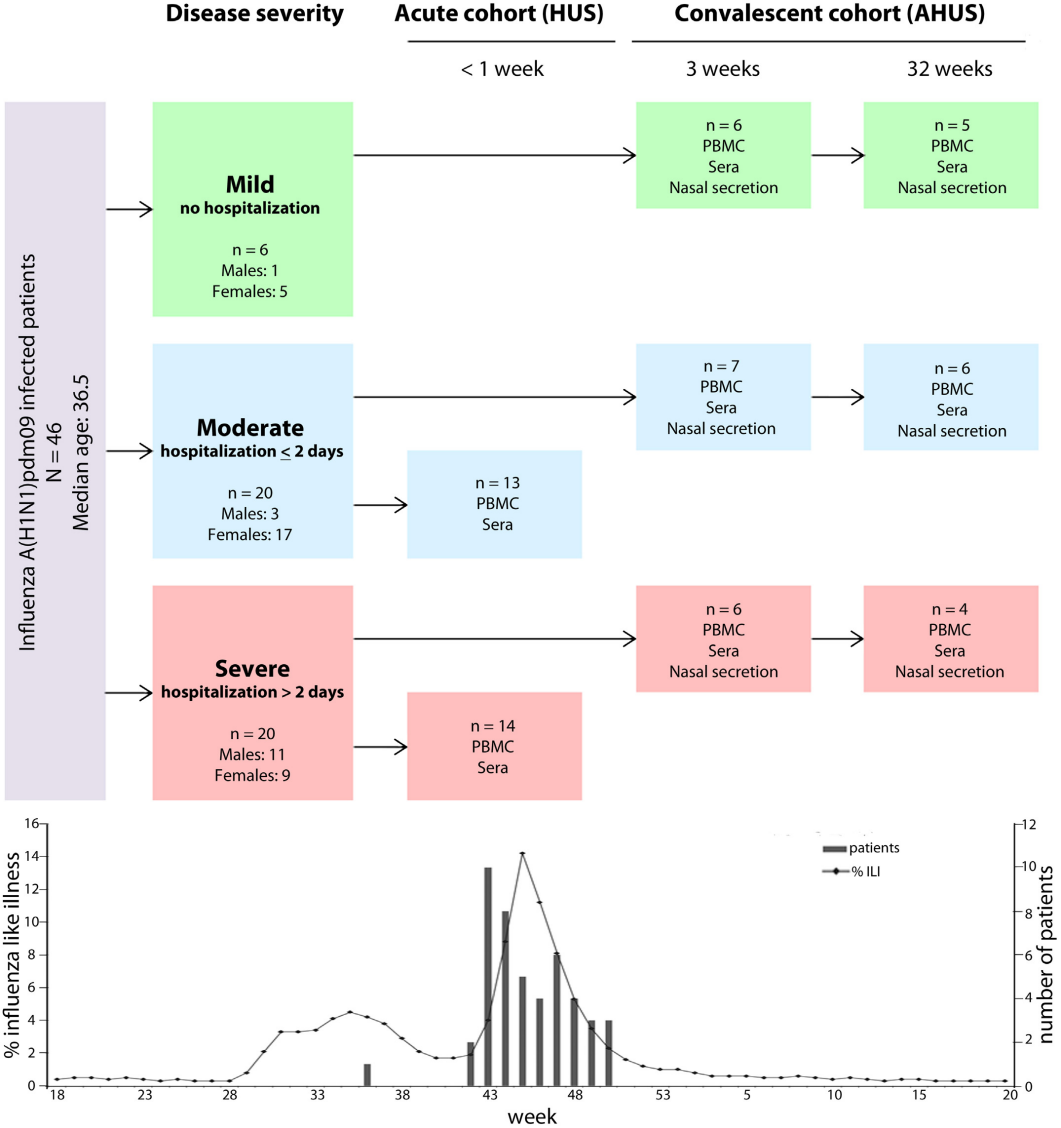
Study design. The inclusion of patients, time and types of samples collected in this study are shown in this flow-chart. Patients were recruited from two Norwegian university hospitals; Haukeland (HUH) or Akershus (AHUS). Nasopharyngeal swabs for influenza A(H1N1)pdm viral RT-PCR diagnosis were collected from patients at the time of inclusion for routine diagnosis. Subsequently sera and peripheral blood mononuclear cells (PBMC) were collected from hospitalized patients at HUH in the acute phase of disease. Sera and PBMC were collected at 3 and 32 weeks post disease onset from patients at AHUS (hospitalized and out-patients). Sample types available from individual patients are described in S1 Table. Sufficient numbers of PBMC were not available for all analyses. Patients were grouped according to disease severity as mild (no hospitalization), moderate (hospitalization ≤ 2 days) and severe (>2 days hospitalization) [12]. The trend-line in the graph below shows people with influenza like illness in Norway from end of April 2009 to May 2010. The first wave was due to other respiratory viruses than pandemic H1N1. In the same graph the number and time of inclusion of patients in this study are shown as columns.

We conducted a prospective observational study in 46 adult patients (>15 years old) during the main wave of influenza A(H1N1)pdm pandemic in October/November 2009 in Norway. Two groups of patients were recruited from two Norwegian university hospitals (Haukeland (HUS) and Akershus (AHUS) university hospital). Nasopharyngeal swabs were collected from patients at inclusion in the study, for confirmatory viral diagnosis by real-time RT PCR. Acute, patients (n = 27) hospitalized >24 hours at HUS provided one blood sample (peripheral blood mononuclear cells (PBMC) and serum) in the acute phase of disease (Fig 1). Convalescent patients (n = 19) were diagnosed initially at AHUS and blood samples were collected in the convalescent phase at 3 and 32 weeks post-infection at a designated outpatient clinic (Fig 1). Patient data were grouped according to disease severity into mild (no hospitalization), moderate (hospitalization ≤ 2 days) and severe illness (hospitalization > 2 days, often with lung infiltrations and oxygen requirement) [12] (S1 Table). All patients met the modified clinical case definition for A(H1N1)pdm09 disease, described previously [12]. Nineteen convalescent patients with influenza like illness (ILI) symptoms were recruited from hospitalized and outpatients, with mild, moderate or severe disease, after RT-PCR confirmation of A(H1N1)pdm09.

The Regional Ethical Committees approved the study (Regional Ethics Committee South-East Norway 2009/1853 and Regional Ethics Committee Western Norway 2009/2295). Written informed consent was obtained upon inclusion.

### Isolation of PBMC and sera

PBMC were separated immediately by density gradient centrifugation of cell preparation tubes (CPT) or from ETDA tubes through Lymphoprep^™^ (StemCell technologies) according to the manufacturer’s instructions and stored in liquid nitrogen in 90% foetal calf serum (FCS)/10% DMSO or 25% FCS/10% DMSO. Serum samples were aliquoted and stored at -20°C.

### Hemagglutination inhibition test

Sera were pre-treated with receptor destroying enzyme (RDE) (Seiken, Japan) [31]. HI antibodies were detected in sera with 0.7% turkey red blood cells and 8 HA units of influenza A/California/07/09(H1N1) virus. HI titers are expressed as the reciprocal of the last serum dilution inhibiting hemagglutination. Negative titers were assigned a value of 5 for calculation purposes.

### Microneutalization assay

Microneutralization (MN) was carried out as described in the WHO protocol [32] with minor modifications. Inactivated sera were pre-incubated with A/California/07/09 like virus. Cells were fixed in methanol containing 0.6% H_2_O_2_ for 20 min. Detection of influenza infected cells was done by ELISA using monoclonal anti-influenza A nucleoprotein primary antibody diluted PBS, 5% skimmed milk powder and 0.1% Tween 20 and Horseradish peroxidase-labeled rabbit anti-mouse IgG secondary antibody diluted PBS, 5% skimmed milk powder, 0.1% Tween 20 and 1% bovine serum albumin. Liquid TMB substrate was used and the reaction was stopped after 18 minutes using 0.5M HCl. The absorbance was measured at 450 and 620nm and the 620nm readings were subtracted from the 450nm readings.

### IgG ELISA

ELISA was used to measure influenza specific IgG in serum [33]. Ninety-six-well plates (maxisorb, Nunc) were coated with 4μg/ml of inactivated virus solution (100μl/well) and allowed to bind for ≥2 days at 4°C [34]. Specific antibody concentrations (arbitrary units) in unknown samples were determined based on defined pools of human sera.

### Intracellular cytokine staining

Frozen PBMC were thawed and rested overnight in RPMI 1640 medium containing L-glutamine, 0.1 mM non-essential amino acids, 10 mM Hepes pH 7.4, 1 mM sodium pyruvate, 100 IU⁄ml penicillin, 100 μg⁄ml streptomycin, 0.25 μg ⁄ml fungizone, and 10% FCS. Lymphocytes were stimulated for 16 hours with A/California/07/09 split virus antigen (2.5μg/ml HA, kindly provided by GSK, Belgium) and anti-CD28 (1μg/ml) anti-CD49d (1μg/ml) antibodies (Pharmingen, USA), Brefeldin A (1μg/ml) and Monensin (0.7μg/ml) (BD Biosciences, USA) [35]. For each patient sample, cytokine levels in non-stimulated cells (cells incubated in lymphocyte medium containing anti-CD28 (1μg/ml) anti-CD49d (1μg/ml) antibodies, Brefeldin A (1μg/ml) and Monensin (0.7μg/ml)) were subtracted from cytokine levels observed in corresponding influenza-stimulated cells, in order to determine the influenza-specific responses. HA-specific cells were stained for CD3, CD4, IFN-γ, IL-2 and TNF-α and analysed by a BD LSRFortessa flow cytometer (acquiring ≥3x10^5^cells). Representative gating strategy is illustrated in S1 Fig.

### IFN-gamma ELISpot assay

IFN-γ responses were measured in pre-coated 96-well plates (Mabtech AB, Sweden) using 200,000 PBMC in AIM-V medium (Gibco) [31]. Positive (anti-CD3 or ConA) and negative controls (DMSO/AIM-V), A/California/07/09 whole virus, split virus antigen and peptide libraries (final concentration of 2μg/ml in DMSO/AIM-V) were added. Peptide assays were conducted for a subset of patients with available PBMCs (n = 16, 11 acute and 5 convalescent patients). The plates were read using a CTL S 6 Ultra V Immunospot analyzer (Cellular Technology Limited, OH, USA), and the results were plotted using GraphPad Prims 5 software (GraphPad Software, Inc., USA).

### Peptide libraries

Synthetic peptide sets have been compiled in order to assess T-cell responses against different influenza antigens (internal or envelope) targeted by different T-cell subsets (CD4 or CD8) [36]. Experimentally verified T-cell epitopes were identified based on querying the Immune Epitope Database [37]. Universal epitopes were selected from influenza strains circulating between 1934–2009 according to prevalence, conservancy, and HLA super type coverage [27]. This approach circumvents the need for individual HLA typing. Peptides unique for A(H1N1)pdm09 have also been identified [36]. The peptide lengths were 8–11 amino acids for MHC class I and ≥ 13 amino acids for MHC class II. The peptides (31 MHC class I and 33 MHC class II) were chemically synthesized by Fmoc chemistry and HPLC purified (Mimotopes, Australia). The peptides were pooled into 7 distinct sets according to strain specificity (universal or pandemic), T-cell subset (CD4/CD8 T-cells), and antigen source (internal or external) [36].

### Statistics

Statistical analyses were performed in GraphPad Prism, version 5 for Mac and Prism 6 for Windows (GraphPad Software, USA). For all statistical tests, a *P-*value of ≤0.05 was considered significant. For comparisons of intracellular cytokine production we used Student’s t-test. All other data were analyzed using non-parametric methods; Mann-Whitney test, Kruskal-Wallis or Wilcoxon test (paired group). Correlations were calculated using Spearman’s correlation coefficient.

## Results

### Study patients enrolled from two major University hospitals

In total 46 patients were enrolled in two separate groups from two university hospitals in Norway during the 2009 pandemic (Fig 1). The patients had a median age of 36.5 years old (age range 19–93), and a female/male ratio of 31/15 (S1 Table). Laboratory samples, epidemiological and demographic data were collected from the two groups of patients: either from acute hospitalized (n = 27, sampled once), or convalescent patients (n = 19, sampled twice, at weeks 3 and 32 post infection). Twenty patients had moderate disease (hospitalized ≤ 2 days), and 20 patients had severe influenza disease (hospitalized >2 days). Six convalescent patients had mild influenza disease (no hospitalization) without any underlying disease conditions. Forty percent of the moderately and severely ill patients had comorbidities: 11 had pulmonary or coronary diseases, six patients had other underlying diseases (autoimmunity, malignancy), and five were pregnant, reflecting the diversity of patients hospitalised during the pandemic in Norway. Of these, seven patients were vaccinated with the Pandemrix vaccine (GSK) (S1 Table).

### Serological responses

#### Severely ill patients had higher antibody responses

**Fig 2:**
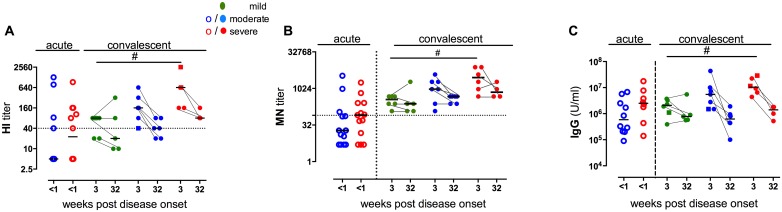
Humoral responses against A(H1N1)pdm09 virus. Humoral responses against A(H1N1)pdm09 virus in acute (one time point) and convalescent patients (3 and 32 weeks following disease onset) plotted according to the disease severity. A) HI titers in acute (n = 27) and convalescent patients (n = 19 and 15). The dotted line represents an HI titer of 40. B) Microneutralization (MN) titers in acute (n = 27) and convalescent patients (n = 19 and 15). The dotted line represents an MN titer of 80. C) Serum IgG concentration in acute (n = 25) and convalescent patients (n = 19 and 15). ○ = acute samples, ● = convalescent samples □ = single samples in the convalescent group. Disease severity is defined as mild (out-patients), moderate (hospitalized ≤ 2 days) or severe (hospitalized > 2 days). ^#^p≤ 0.05 (Mann Whitney test (HI and IgG) unpaired t-test (MN)).

To study the humoral immune response, sera were collected from all patients (one time point for the acute patients and two time points for 15 paired convalescent samples) and tested for A(H1N1)pdm09-specific antibodies with HI, MN and IgG ELISA assay (Fig 2A,2B, and 2C). No data on pre-infection antibody titers were available, as patients were recruited upon clinical presentation with confirmed A(H1N1)pdm09 disease. HI titers ≥40 were present in sera from 11/27 of acute and 17/19 of convalescent patients at three weeks post infection (Fig 2A). Seven patients in the acute group (3 moderately and 4 severely ill) received pandemic vaccination, but only 4 of these patients had HI titers ≥40 (2 moderate and 2 severe). In the convalescent patient group, 2 patients (severely ill) were vaccinated and both had titers ≥ 40 (S1 Table). We did not find differences in HI or MN responses in patients with or without comorbidities.

The convalescent patients recovering from severe disease had significantly higher HI titers (all subject ≥40), MN titers and IgG antibody levels than patients with mild disease at 3 weeks post post-infection (Fig 2A–2C). Similarly, in the acute patients there was a trend that the severely ill patients had higher antibody responses than the moderately ill (Fig 2A–2C). In order to study the functionality of the antibody response, MN assay was performed (Fig 2B). The MN data correlated with the HI results (both patient groups; r = 0.9, p<0.0001). For the convalescent patients the kinetics of the antibody response was investigated in the paired subjects, and the antibody levels correlated significantly with severe and moderate disease (HI-titer: r = 0.786, p = 0.0001 and IgG: r = 0.677, p = 0.0014). The HI and IgG antibody levels declined significantly between 3 and 32 weeks post infection in the convalescent patients, but remained above the protective HI-threshold in most patients (67%), with severely ill patients showing higher antibody responses compared to patients with mild disease. Fig 2A).

### Cellular responses

#### High CD4^+^ T-cell TNF-α response in acute severely ill patients

**Fig 3:**
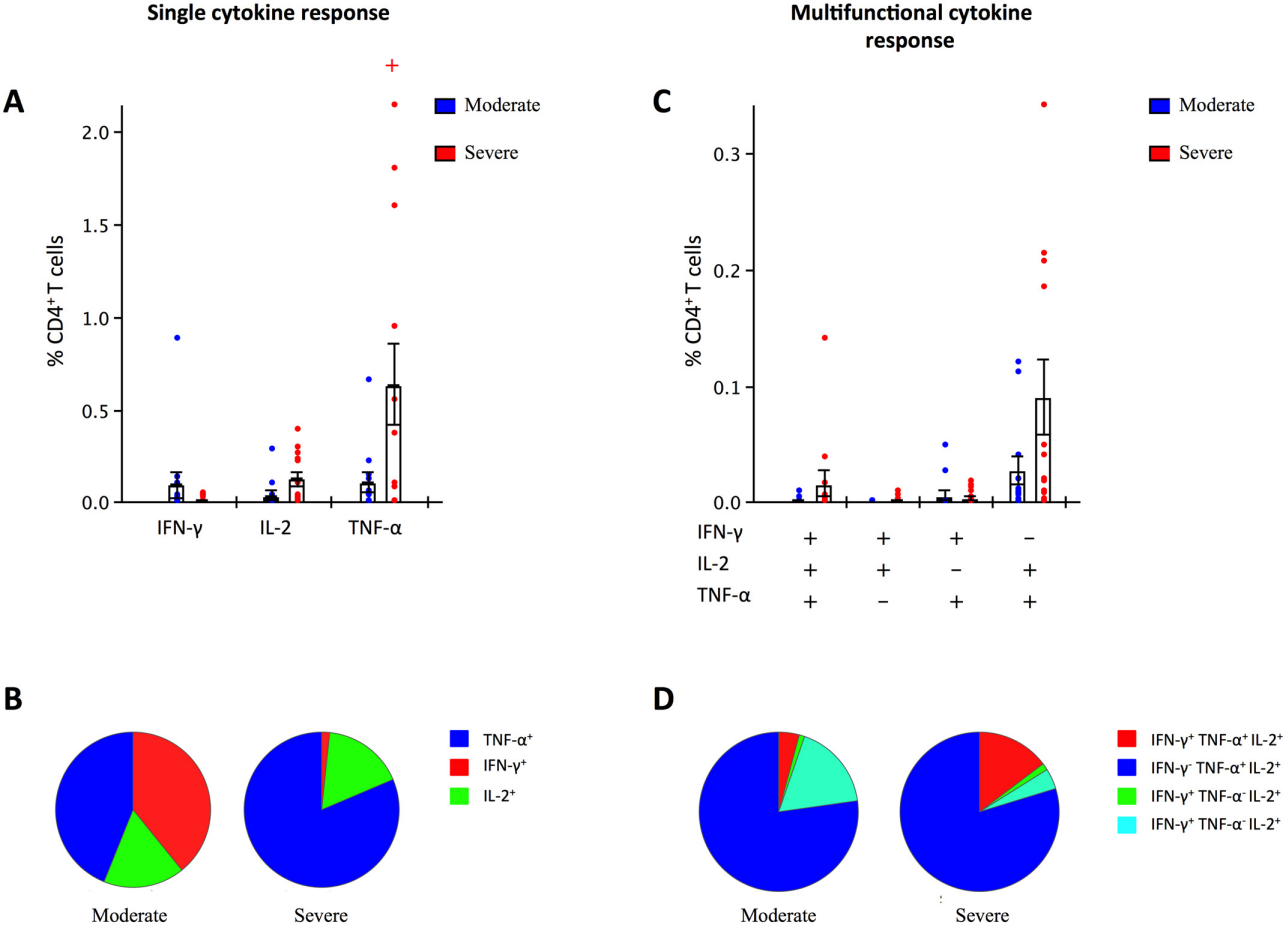
CD4^+^ T-cell cytokine responses in moderately and severely ill acute patients. PBMC from acute patients (n = 24) were stained for intracellular cytokines and the percentage of CD4^+^ T-cells secreting either single (A and B) or multiple (C and D) cytokines were measured by flow cytometry. Each symbol represents the response of one individual with bars depicting the mean and SEM percentage of CD4^+^ T-cells. ^+^p<0.05 (Student’s t test). Gating strategy is shown in S1 Fig. Disease severity is defined as moderate (hospitalized ≤ 2 days) or severe (hospitalized > 2 days).

To study the functionality of T-cell responses, PBMC from acute subjects (n = 24) were stimulated with split virus antigen (predominantly containing surface antigens) and analyzed for intracellular cytokines by flow cytometry. The percentages of CD4^+^ T-cells secreting HA-specific single (Fig 3A and 3B) or multiple cytokines (Fig 3C and 3D) are shown. High unspecific background levels of TNF-α were observed in moderately and severely ill patients, with the highest levels in the severely ill patients (range 0.041–4.12% of CD4^+^ T-cells, data not shown). Severely ill patients (n = 12) had significantly higher numbers of influenza specific T-cells secreting TNF-α than moderately ill patients (n = 12) (Fig 3A and 3B). When comparing the severely ill with moderately ill patients we also observed a non-significant trend with higher levels of IL-2 (mean of 0.12% and 0.04%, respectively) and lower levels of IFN-γ (mean of 0.01% and 0.09% of, respectively) positive CD4^+^ T-cells. The percentage of CD4^+^ T-cells simultaneously expressing two (IL-2/TNF-α) or three (IFN-γ/IL-2/TNF-α) cytokines was higher in the severely ill than in the moderately ill group, although the difference was not significant (Fig 3C and 3D).

#### Acute patients have a high envelope specific-CD4^+^ and low CD8^+^ T-cell response

**Fig 4:**
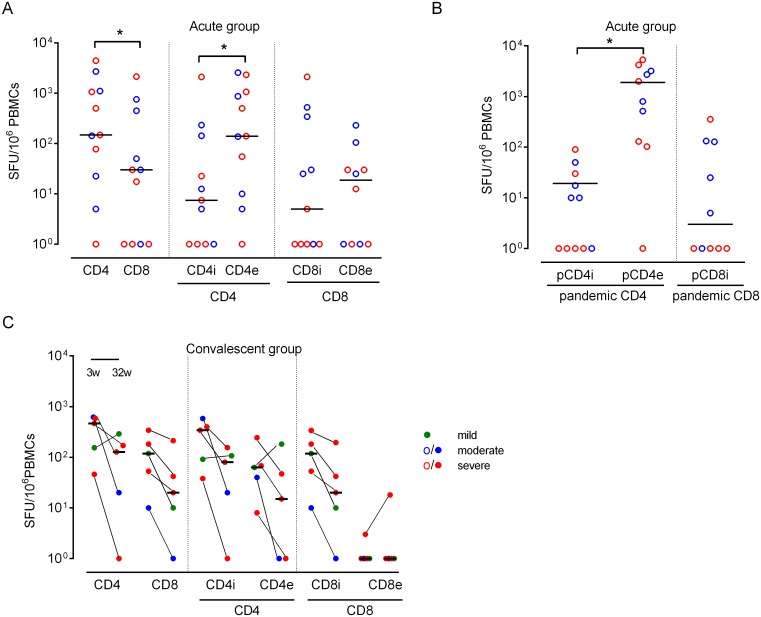
T-cell responses measured by IFN-γ Elispot assay after stimulation with universal or A(H1N1)pdm09 specific peptides. A) T-cell responses against universal influenza epitopes in a subset of acute patients (n = 11). B) T-cell responses against A(H1N1)pdm09 specific epitopes in a subset of acute patients (n = 11). C) T-cell responses against universal influenza epitopes in a subset of convalescent patients (n = 5) with paired samples for 3 and 32 weeks. ○ = acute samples, ● = convalescent samples. Disease severity is defined as mild (out-patients), moderate (hospitalized ≤ 2 days) or severe (hospitalized > 2 days). The bars are plotted as median with range. The T-cell responses were directed against epitopes from internal (i = NP, M1, PA, PB and NS) or external (e = HA and NA) influenza antigens. CD4 = CD4i + CD4e and CD8 = CD8i + CD8e. *p≤ 0.05 (Wilcoxon matched-pair signed-rank test). PBMC were also shown to respond to stimulation with live influenza virus (data not shown).

To further dissect the T-cell responses, PBMC from a subset of acute patients (n = 11) were stimulated with different peptide pools of influenza A specific epitopes in the IFN-γ Elispot assay [36] (Fig 4A). We measured CD4^+^ or CD8^+^ responses induced by epitopes from either universal (envelope or internal) or pandemic specific antigens [36]. We observed significantly higher frequencies of cells within the CD4^+^ compared with CD8^+^ compartment (5-fold) (Fig 4A). The number of CD4^+^ T-cells recognizing epitopes from the viral envelope (HA and NA; CD4e peptides) was significantly higher than CD4^+^ T-cells recognizing the conserved core antigens (PA, PB, M, NP, NS2 and NS1; CD4i peptides) (Fig 4A). Overall, the acute patients were characterized by low CD8^+^ T-cell responses. No significant differences in T-cell responses were found between moderately and severely ill patients (Fig 4A). When PBMC from the acute patients were stimulated with CD4^+^ and CD8^+^ T-cell epitopes unique to the pandemic strain, the CD4^+^ T-cell responses were also predominantly directed against the HA and NA antigens (pCD4e) of the pandemic strain.

#### CD4^+^ and CD8^+^ T-cell responses were directed against internal epitopes in the convalescent patients

In a subset of the convalescent patients paired samples from two time points (3 and 32 weeks) were available (n = 5). In contrast to acute phase patients, similar total CD4^+^ and CD8^+^ T-cell responses were observed at 3 and 32 weeks post-infection (Fig 4C). Influenza specific CD4^+^ and CD8^+^ T-cell responses were higher in the convalescent patients (week 3) as compared with the acute patients (3- or 4-fold, respectively). Moreover, higher frequencies of both CD4^+^ and CD8^+^ T-cells recognized internal antigens compared with envelope antigens at both time points (Fig 4C) (p = 0.063 at week 3 for both CD4^+^ and CD8^+^ T-cells). A clear decline in frequencies of both IFN-γ producing CD4^+^ and CD8^+^ T-cells was observed over time (Fig 4C). Stimulation with live virus showed similar T-cell kinetics as obtained by peptide pools (data not shown). No clear trends linking the T-cell responses to clinical severity could be observed for the convalescent patients, possibly due to the low number of patients in each disease group.

## Discussion

Identifying risk factors and immune correlates of protection against human influenza is crucial for future pandemic preparedness. There is currently limited data on immunological responses in hospitalized patients due to the difficulties of collecting samples in the midst of a pandemic. The emergence of the 2009 influenza pandemic represented a unique opportunity to relate clinical outcomes to influenza specific immune responses. Here, we have characterized both antibody and T-cell responses in two study groups of either acute or convalescent patients with different disease severities (mild, moderate and severe) in Norway. We were particularly interested in studying T-cell responses in severely as compared with moderately ill patients. Limitations in the possibilities to obtain an ideal sample set, including base line and follow up samples from the same patients have to be taken into consideration when interpreting the results. Nevertheless, the patients in our study reflect the pandemic situation in Norway with respect to age, comorbidities and vaccination status, resulting in different pre-existing immunity status.

Patients were included upon infection and therefore we unfortunately do not have data on pre-infection antibody levels in our patients. However, Norwegian seroprevalence data showed low frequencies of pre-existing protective antibodies (1,7%) in the population prior to the 2009 pandemic as well as in health care workers [38,39]. Therefore the antibodies measured in our patients are probably induced by infection with the pandemic virus. Only four of the seven vaccinated patients in the acute group had high antibody levels, which may have been influenced by the timing of pandemic vaccination in Norway since the pandemic wave, and vaccination occurred almost simultaneously [40].

Antibody responses were influenced by disease severity with the highest levels of antibodies found in patients with severe disease. Although the antibody levels declined 8 months after infection, protective antibody levels remained present in patients with severe disease. This is in agreement with a recent study showing a correlation between the severity of influenza disease and antibody levels [41]. Another study from the UK showed high antibody titers for a minimum of 15 months after natural infection with A(H1N1)pdm09 [31]. It has previously been shown that severely ill patients had elevated levels of viral replication in the lower respiratory tract or extended viral shedding, which might lead to high antibody levels [42]. No measurable differences in upper respiratory tract viral loads were found between the different illness severities in our patients when comparing PCR data at the time of inclusion (Ct values in S1 Table). Two patients with mild disease did not achieve protective HI antibody levels, confirming previous findings that not all infected individuals show HI seroconversion [43].

Pandemic influenza may occasionally cause viremia in severely ill or immunocompromised patients [44–46]. Four of the 27 hospitalized patients in the acute group with moderate or severe disease had viremia, with systemic viral dissemination. Viral factors, in particular a point mutation in HA, have been associated with severe disease [16,17]. No such mutation was detected in the viruses sequenced from our PCR positive patients (S2 Table). However, *de novo* mutations may occur and a low frequency of mutant virus in a high background of 222D virus may not be detected in the upper respiratory tract. Several patients with clinical pandemic influenza were PCR negative, possibly with lower respiratory tract infection not detected by nasopharyngeal swabs. Therefore we cannot exclude that this mutation may have been present in the lower respiratory tract of these patients [44,47,48].

T-cells are central in coordinating the immune response, and both the magnitude and the quality of T-cell responses are critical for the control of viral infections [42,49]. The high unspecific TNF-α background levels observed in the severely ill patients in this study, suggests an overall elevated and possibly dysregulated immune activation in these individuals. Moreover, the high frequency of single TNF-α CD4^+^ T-cells in the severely ill patients suggest exhaustion or an altered immune response in these patients, affirmed by low single IFN-γ CD4^+^ T-cells and low frequencies of multifunctional cells [50]. Protective responses to respiratory viruses are typically biased towards a Th1 response, producing high levels of IFN-γ, but also IL-2 and TNF-α, promoting cytolytic activity and viral clearance [51,52]. The multifunctional CD4^+^ T-cells are regarded as functionally superior to single-cytokine producers [53]. The low levels of multifunctional CD4^+^ T-cells in the acute, severely ill patients could possibly be linked to the severity of their disease. However, future studies with higher numbers of participants are needed to confirm this. Although present at low frequencies, the multifunctional HA specific CD4^+^ T-cells were dominated by IFN-γ^-^IL2^+^TNF-α cells. This is in agreement with previous findings that after A(H1N1)pdm09 exposure, a primed IFN-γ^-^IL2^+^TNF-α^+^ non-polarized precursor T-cell population (Thpp) has been observed [54], representing a recently induced memory response to influenza [55]. This small multifunctional CD4^+^ T-cell population has high proliferative potential and may be important for protection against future infection [54]. The general elevated T-cell responses in the convalescent group may reflect recall expansion of this subpopulation.

After natural A(H1N1)pdm09 infection CD4^+^ T-cells recognize both unique and conserved HA epitopes [56]. HA specific naïve T-cells undergo significant expansion, whereas memory T-cells directed towards conserved epitopes have a more restricted expansion [56]. This may be reflected in the striking dominance of T-cell responses against external envelope antigens seen in the acute patients as opposed to conserved internal antigens in the convalescent patients.

Recent studies suggest that different immune memory profiles may develop depending on the severity of A(H1N1)pdm09 infection [28,29]. In agreement with this, we found significant differences in T-cell responses between and within our patient groups. Acute patients showed higher CD4^+^ than CD8^+^ T-cell responses compared to the healthy Norwegian population (5-fold /1.5-fold respectively) [36]. The convalescent patients showed higher levels of both CD4^+^ and CD8^+^ T-cells, compared to acute patients and healthy individuals. However, no significant differences were found between disease severities.

Although pre-existing cross-reactive CD8^+^ T-cells have been correlated with reduced severity of symptoms during natural influenza infection [26], it is not clear whether these circulating cells protect against disease or whether they potentially reflect local pulmonary T-cells that mediate viral clearance [57]. The low levels of peripheral CD8^+^ T-cells may be linked to disease severity. However, the low CD8^+^ levels may be due homing of this T-cell subset to the infected lung tissue, and hence absence in the blood. In support of this, Zhao and coworkers found that high levels of peripheral CD4^+^ T-cells against internal viral proteins in the early phase of infection, rather than low levels of CD8^+^ cells T-cells, correlated with disease severity during the 2009 pandemic [28]. The higher levels of both CD4^+^ and CD8^+^ T-cells in the convalescent patient group could indicate subsequent proliferation in the blood. The phenotype of T-cells protecting against influenza infection remains to be defined. While our T-cell data provide preliminary evidence for the importance of T-cells in protection from severe disease, future studies should focus on the functionality of these T-cells (killing and degranulation markers) to dissect how T-cells influence disease severity.

Excessive immune responses have been assumed to play a role in the pathogenesis of influenza virus disease, this assumption has been challenged by the findings that severe disease is characterized by inadequate, rather than excessive, adaptive immune responses and robust viral replication [57]. Our data suggests that both the phenotype of T-cells and the influenza epitopes they target vary according to the phase of infection. However, additional studies, following larger cohorts of well-characterized influenza infected individuals will be necessary to define the relationship between T-cell subpopulations and disease severity or phase of infection.

Despite the limitations of this study, the apparently low levels of CD8^+^ T-cell responses in patients hospitalized during the acute phase, suggests an important role of these T-cells in protective immunity against influenza. Moreover, the observation that both CD4^+^ and CD8^+^ T-cell responses are directed against epitopes from conserved internal antigens in the convalescent phase of infection may guide universal influenza vaccine development. Our results support the idea that the clinical severity of pandemic infection is influenced by the host´s immune response and not only the characteristics of the novel virus.

## Supporting Information

S1 Fig Representative gating strategy for unstimulated and influenza-specific intracellular cytokine secretion analysis by flow cytometry. Frozen PBMC form a severely ill acute patient were thawed and rested overnight and A) incubated for 16 hours (5%CO_2_, 37°C) in lymphocyte medium containing anti-CD28 (1μg/ml) antibodies, Brefeldin A(1μg/ml) and Monensin (0.7μg/ml) or B) stimulated for 16 hours with 2.5μg/ml HA of A/California/07/09 split vius vaccine and anti-CD28 (1μg/ml) anti-CD49d (1μg/ml) antibodies, Brefeldin A(1μg/ml) and Monensin (0.7μg/ml). The basal cytokine levels in non-stimulated cells were subtracted from the cytokine levels observed in the influenza stimulated cells. Cells were stained with flurochrome conjugated antibodies against CD3, CD4, IFN-γ, IL-2 and TNF-α and acquired by a BD LSRFortessa flow cytometer (acquiring ≥3x10^5^cells per sample) and data analyzed by FloJo software (Version 8.8.7).
(TIFF)

S1 Methods and Results . (DOCX)

S1 Table Demographics of the patients included in the study. ^1^Disease severity is defined as mild (out-patients), moderate (hospitalized ≤ 2 days) or severe (hospitalized > 2 days).
^2^Time from onset of clinical symptoms [12].
^3^PBMCs: + samples included in analyses, (+) samples excluded from analyses,—samples never received for analyses
^4^HI titer only.
^5^For convalescent patients, the HI titers are given as: titer at 3 weeks (titer at 32 weeks), e.g. 160 (20).
(DOCX)

S2 Table Overview of the mutations found in the HA gene. (DOCX)
