# Supplementary figures and images for: Immune Responses in Acute and Convalescent Patients with Mild, Moderate and Severe Disease during the 2009 Influenza Pandemic in Norway
Source: PLoS One. 2015 Nov 25;10(11):e0143281. doi: 10.1371/journal.pone.0143281 (PMC4659565; doi:10.1371/journal.pone.0143281)

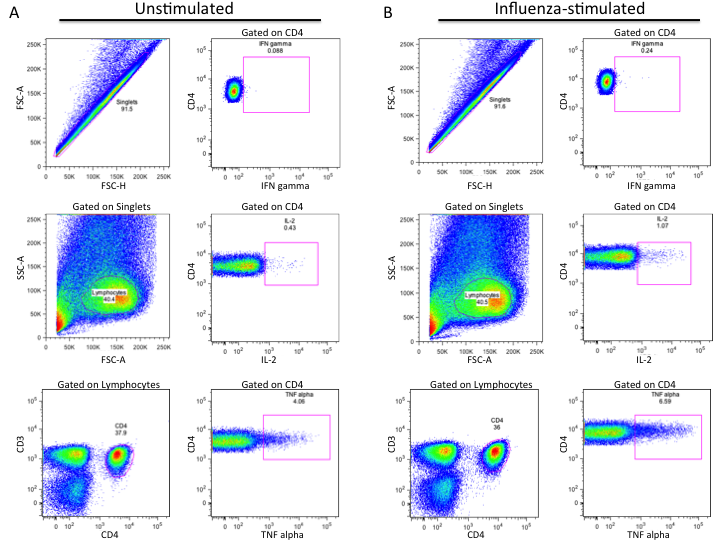

Supplement: S1 Fig — Frozen PBMC form a severely ill acute patient were thawed and rested overnight and A) incubated for 16 hours (5%CO2, 37°C) in lymphocyte medium containing anti-CD28 (1μg/ml) antibodies, Brefeldin A(1μg/ml) and Monensin (0.7μg/ml) or B) stimulated for 16 hours with 2.5μg/ml HA of A/California/07/09 split vius vaccine and anti-CD28 (1μg/ml) anti-CD49d (1μg/ml) antibodies, Brefeldin A(1μg/ml) and Monensin (0.7μg/ml). The basal cytokine levels in non-stimulated cells were subtracted from the cytokine levels observed in the influenza stimulated cells. Cells were stained with flurochrome conjugated antibodies against CD3, CD4, IFN-γ, IL-2 and TNF-α and acquired by a BD LSRFortessa flow cytometer (acquiring ≥3x10^5^cells per sample) and data analyzed by FloJo software (Version 8.8.7). (TIFF) [file pone.0143281.s001.tiff]
