# Supplementary material for: Immune Responses in Acute and Convalescent Patients with Mild, Moderate and Severe Disease during the 2009 Influenza Pandemic in Norway
Source: PLoS One. 2015 Nov 25;10(11):e0143281. doi: 10.1371/journal.pone.0143281 (PMC4659565; doi:10.1371/journal.pone.0143281)
Supplement: S1 Methods and Results — (DOCX) [file pone.0143281.s002.docx]

**Supplementary Methods**

*Influenza virus characterization*

The entire HA gene, or the region of HA covering amino acid position 222 (D/E/G/N), associated with receptor-binding specificity and disease severity [[1](#_ENREF_1)], was sequenced from reverse transcribed viral RNA from 18 patients, according to standard procedures [[2](#_ENREF_2), [3](#_ENREF_3)]. Detection of viral RNA was performed in nucleic acid extract from blood and nasopharyngeal swabs. Viral loads were measured by RT-PCR (Ct values <15 - >40) [[3](#_ENREF_3)] (Supplementary Table 1).

**Supplementary Results**

*No differences in viral loads between severe and moderate patients*

Patients provided a nasopharyngeal swab upon contact with healthcare services with suspected pandemic influenza. Nasopharyngeal swabs were positive for H1N1pdm09 by RT-qPCR in 30 patients included in the study, with a mean Ct value of 27.5 for confirmed influenza A positive patients by RT-PCR) (S1 Table). Four of the 27 acute patients (15.4%) had viremia, two patients with moderate and two with severe influenza disease. No statistical significant differences were observed in viral loads in the upper respiratory tract between the mild, moderate, or severe patient groups. Substitutions from D to E/G/N at position 222 have been associated with increased disease severity [[1](#_ENREF_1)]. The complete sequence of the viral HA gene was successfully obtained for 6 patients in the acute group, and partial sequence was obtained for 12 patients in the convalescent group. We found several amino acid mutations, however none of these have previously been linked to disease severity (S2 Table).

1. Baldanti F, Campanini G, Piralla A, et al. Severe outcome of influenza A/H1N1/09v infection associated with 222G/N polymorphisms in the haemagglutinin: a multicentre study. Clin Microbiol Infect 2011;17:1166-9

2. CDC, The WHO Collaborating Centre for influenza at CDC Atlanta USoA. Sequencing primers and protocol WHO protocol 2009

3. Brittain-Long R, Nord S, Olofsson S, Westin J, Anderson LM and Lindh M. Multiplex real-time PCR for detection of respiratory tract infections. J Clin Virol 2008;41:53-6
