# Supplementary material for: Immune Responses in Acute and Convalescent Patients with Mild, Moderate and Severe Disease during the 2009 Influenza Pandemic in Norway
Source: PLoS One. 2015 Nov 25;10(11):e0143281. doi: 10.1371/journal.pone.0143281 (PMC4659565; doi:10.1371/journal.pone.0143281)
Supplement: S1 Table — 1Disease severity is defined as mild (out-patients), moderate (hospitalized ≤ 2 days) or severe (hospitalized > 2 days). 2Time from onset of clinical symptoms [12]. 3PBMCs: + samples included in analyses, (+) samples excluded from analyses,—samples never received for analyses 4HI titer only. 5For convalescent patients, the HI titers are given as: titer at 3 weeks (titer at 32 weeks), e.g. 160 (20). (DOCX) [file pone.0143281.s003.docx]

**Supplementary Table 1: Patients included in the study**

| Patient ID | Disease grade^1^ | Underlying condition | Time point of blood sample collection^2^ | | | Material available/included in the figures | | Pandemrix vaccination | HI titer^5^ | Cp values  in RT-PCR^6^ |
| --- | --- | --- | --- | --- | --- | --- | --- | --- | --- | --- |
|  |  |  | Acute | Sampling 1 (~3 w) | Sampling 2 (~32 w) | Serum | PBMC^3^  (no of time points) |  |  |  |
| 2-1 | Mild | None | - | + | - | + | - | - | 80 | 28.2 |
| 2-2 | Mild | None | - | + | + | + | - | - | 20 (10) | 23.4 |
| 2-3 | Mild | None | - | + | + | + | + | - | 80  (320) | 18.3 |
| 2-4 | Mild | None | - | + | + | + | + | - | 80 (80) | 33.8 |
| 2-14 | Mild | None | - | + | + | + | + (2) | - | 20 (10) | 22.5 |
| 2-16 | Mild | None | - | + | + | + | + | - | 80 (20) | 33.2 |
| 1-1 | Moderate | None | + | - | - | + | - | + | 5 | 32.3 |
| 1-3 | Moderate | None | + | - | - | + | - | - | 5 | 33.2 |
| 1-4 | Moderate | None | + | - | - | + | (+)+ | - | 783.8 | - |
| 1-10 | Moderate | Pregnancy | + | - | - | + | - | - | 5 | - |
| 1-12 | Moderate | None | + | - | - | + | + | - | 5 | - |
| 1-15 | Moderate | None | + | - | - | + | (+)+ | - | 5 | n.a. |
| 1-16 | Moderate | Chronic gastrointestinal disease | + | - | - | + | (+)+ | + | 5 | - |
| 1-22 | Moderate | None | + | - | - | + | (+) | - | 83.2 | 32.8 |
| 1-30 | Moderate | Chronic liver disease | + | - | - | + | (+) | + | 1280 | 35.8 |
| 1-35 | Moderate | Chronic pulmonary disease | + | - | - | + | - | + | 40 | - |
| 1-45 | Moderate | Pregnancy | + | - | - | + | - | - | 5 | - |
| 1-47 | Moderate | Chronic pulmonary disease | + | - | - | + | - | - | 5 | 31.2 |
| 1-57 | Moderate | None | + | - | - | + | (+)+ | - | 5 | - |
| 2-5 | Moderate | None | - | + | + | + | + | - | 80 (20) | 16.3 |
| 2-6 | Moderate | None | - | + | + | + | + | - | 160 (40) | 32 |
| 2-7 | Moderate | None | - | + |  | + | + | - | 40 | 19.6 |
| 2-8 | Moderate | None | - | + | + | + | + | - | 160 (20) | 36.7 |
| 2-9 | Moderate | Pregnancy | - | + | + | + | + | - | 320 (80) | 29.2 |
| 2-10 | Moderate | Pregnancy | - | + | + | + | + (2) | - | 160 (40) | 24.9 |
| 2-12 | Moderate | Malignancy | - | + | + | + | + | - | 640 (80) | 17.9 |
| 1-2 | Severe | Autoimmune disease | + | - | - | + | - | - | 5 | 29.7 |
| 1-9 | Severe | Asthma. hypertension | + | - | - | +^4^ | + | - | 5 | - |
| 1-18 | Severe | None | + | - | - | +^4^ | (+)+ | - | 923 | - |
| 1-24 | Severe | Chronic heart condition | + | - | - | + | - | - | 5 | - |
| 1-25 | Severe | None | + | - | - | + | (+) | - | 5 | - |
| 1-29 | Severe | None | + | - | - | + | + | - | 104.8 | - |
| 1-37 | Severe | None | + | - | - | + | + | + | 5 | 35.2 |
| 1-39 | Severe | Earlier Pneumonia | + | - | - | + | - |  | 160 | - |
| 1-44 | Severe | Malignancy | + | - | - | + | - | + | 160 | 36.4 |
| 1-42 | Severe | None | + | - | - | + | - | - | 5 | - |
| 1-50 | Severe | Chronic heart condition | + | - | - | + | + | + | 160 | - |
| 1-51 | Severe | None | + | - | - | + | + | - | 40 | - |
| 1-53 | Severe | Chronic heart condition. Diabetes, | + | - | - | + | - | - | 5 | - |
| 1-54 | Severe | Chronic heart condition | + | - | - | + | - | - | 80 | 30.7 |
| 2-11 | Severe | Asthma | - | + | + | + | + (2) | - | 160 (80) | 14.8 |
| 2-13 | Severe | Pregnancy | - | + | - | + | + | - | 640 | 32.6 |
| 2-15 | Severe | None | - | + | - | + | - | - | 2560 | 21.7 |
| 2-17 | Severe | None | - | + | + | + | + (2) | - | 640 (160) | 32.8 |
| 2-18 | Severe | Kidney transplantation | - | + | + | + | + | + | 160 (80) | 27.1 |
| 2-19 | Severe | Asthma | - | + | + | + | + (2) | + | 640 (80) | 17.6 |

^1^Disease grade defined as: Mild: out-patients, Moderate: hospitalized ≤ 2 days, Severe: hospitalized > 2 days

^2^Time from onset of clinical symptoms.

^3^PBMCs: + samples included in analyses, (+) samples excluded from analyses, - samples never received for analyses

^4^HI titer only.

^5^For convalescent patients, the HI titers are given as: titer at 3 weeks (titer at 32 weeks), e.g. 160 (20)

^6^”-“: no influenza A virus detected by RT-PCR at detection threshold of 40 cycles
