# Supplementary material for: Immune Responses in Acute and Convalescent Patients with Mild, Moderate and Severe Disease during the 2009 Influenza Pandemic in Norway
Source: PLoS One. 2015 Nov 25;10(11):e0143281. doi: 10.1371/journal.pone.0143281 (PMC4659565; doi:10.1371/journal.pone.0143281)
Supplement: S2 Table — (DOCX) [file pone.0143281.s004.docx]

Supplementary Table 1

|  |  |  |  | |  | amino acid position (HA) | | | | | | | | | | | | | | | |  |
| --- | --- | --- | --- | --- | --- | --- | --- | --- | --- | --- | --- | --- | --- | --- | --- | --- | --- | --- | --- | --- | --- | --- |
| Isolate | 32 | 83 | 103 | 125 | 128 | 162 | 198 | 203 | 222 | 223 | 230 | 232 | 256 | 271 | 272 | 314 | 321 | 372 | 374 | 411 | 456 | |
| California/07/09 (FJ969540) | L | P | E | N | S | S | Y | S | X | X | Y | T | A | P | V | L | I | I | E | V | R | |
| 1-30 |  | S |  |  |  |  |  | T | D | Q |  |  |  |  |  |  | V |  |  |  |  | |
| 1-1 |  | S |  |  |  |  |  | T | D | Q |  |  |  |  |  |  | V |  |  |  |  | |
| 1-2 |  | S |  |  |  |  |  | T | D | Q |  |  |  |  |  |  | V |  |  |  |  | |
| 1-47 |  | S |  |  |  |  |  | T | D | Q |  |  |  |  |  |  | V |  |  |  |  | |
| 1-15 |  | S |  |  |  |  |  | T | D | Q | H |  |  |  |  |  | V |  |  |  |  | |
| 1-54 |  | S |  |  |  |  |  | T | D | Q |  |  |  |  |  |  | V |  | K |  |  | |
| 2-2 | - | - | - | - | - | - | - | T | D | Q |  |  |  |  |  |  |  | - | - | - | - | |
| 2-3 | - | - | - | - | - | - | - | T | D | Q |  |  |  |  |  |  |  | - | - | - | - | |
| 2-5 | - | - | - | - | - | - | - | T | D | Q |  |  |  |  |  |  |  | - | - | - | - | |
| 2-7 | - | - | - | - | - | - | - | T | D | Q |  |  |  |  |  |  |  | - | - | - | - | |
| 2-9 | - | - | - | - | - | - | - | T | D | Q |  |  |  |  |  |  | V | - | - | - | - | |
| 2-10 | - | - | - | - | - | - | - |  | D | Q |  |  |  |  |  |  | V | - | - | - | - | |
| 2-11 | - | - | - | - | - | - | - | T | D | Q |  |  |  | Q |  |  |  | - | - | - | - | |
| 2-12 | - | - | - | - | - | - | - | T | D | Q |  |  |  |  |  |  |  | - | - | - | - | |
| 2-14 | - | - | - | - | - | - | - | T | D | Q |  |  | T |  | A |  |  | - | - | - | - | |
| 2-15 | - | - | - | - | - | - | - | T | D | Q |  |  |  |  |  |  |  | - | - | - | - | |
| 2-18 | - | - | - | - | - | - | - | T | D | Q |  |  |  |  |  |  |  | - | - | - | - | |
| 2-19 | - | - | - | - | - | - | - | T | D | Q |  |  |  |  |  |  |  | - | - | - | - | |

-: Not Determined.
